# *PIM1*-minicircle as a therapeutic treatment for myocardial infarction

**DOI:** 10.1371/journal.pone.0173963

**Published:** 2017-03-21

**Authors:** Nan Liu, Bingyan J. Wang, Kathleen M. Broughton, Roberto Alvarez, Sailay Siddiqi, Rebeca Loaiza, Nicky Nguyen, Pearl Quijada, Natalie Gude, Mark A. Sussman

**Affiliations:** Biology Department, San Diego State University, San Diego, California, United States of America; Academia Sinica, TAIWAN

## Abstract

*PIM1*, a pro-survival gene encoding a serine/ threonine kinase, influences cell proliferation and survival. Modification of cardiac progenitor cells (CPCs) or cardiomyocytes with *PIM1* using a lentivirus-based delivery method showed long-term improved cardiac function after myocardial infarction (MI). However, lentivirus based delivery methods have stringent FDA regulation with respect to clinical trials. To provide an alternative and low risk *PIM1* delivery method, this study examined the use of a non-viral modified plasmid-minicircle (MC) as a vehicle to deliver *PIM1* into mouse CPCs (mCPCs) *in vitro* and the myocardium *in vivo*. MC containing a *turbo gfp* reporter gene (*gfp*-MC) was used as a transfection and injection control. *PIM1* was subcloned into *gfp*-MC (*PIM1*-MC) and then transfected into mCPCs at an efficiency of 29.4±3.7%. *PIM1*-MC engineered mCPCs (*PIM1*-mCPCs) exhibit significantly (P<0.05) better survival rate under oxidative treatment. *PIM1*-mCPCs also exhibit 1.9±0.1 and 2.2±0.2 fold higher cell proliferation at 3 and 5 days post plating, respectively, as compared to *gfp*-MC transfected mCPCs control. *PIM1*-MC was injected directly into ten-week old adult FVB female mice hearts in the border zone immediately after MI. Delivery of *PIM1* into myocardium was confirmed by GFP^+^ cardiomyocytes. Mice with *PIM1*-MC injection showed increased protection compared to *gfp*-MC injection groups measured by ejection fraction at 3 and 7 days post injury (P = 0.0379 and P = 0.0262 by t-test, respectively). Success of *PIM1* delivery and integration into mCPCs *in vitro* and cardiomyocytes *in vivo* by MC highlights the possibility of a non-cell based therapeutic approach for treatment of ischemic heart disease and MI.

## Introduction

Heart disease continues to be the top cause of adult mortality worldwide [1], with estimated 610,000–760,000 deaths annually in 2000–2010 [2, 3]. Myocardial Infarction (MI), leading to irreversible death of affected heart muscle, is one of the major players contributing to increased global mortality. Traditional therapies focus on attenuating the remodeling of the heart after MI and sustaining viable heart function [4, 5]. Emerging therapies include adoptive transfer of stem cells post MI in patients with heart disease [6–8]. One such successful stem cell clinical trial used cardiac progenitor cells (CPCs) in patients suffering from ischemic cardiomyopathy [6]. CPCs are lineage (CD5, CD45R (B220), CD11b, Anti-Gr-1 (Ly-6G/C), 7–4, and Ter-119) negative and stem cell growth factor receptor (c-KIT) positive (LIN^-^c-KIT^+^) cells that exist within the mammalian heart [9]. LIN^-^c-KIT^+^ mouse cardiac progenitor cells (mCPCs) show self-renewal potential and the ability to differentiate into CMs, smooth muscle cells and endothelial cells *in vitro* [9, 10] and promote myocardial repair *in vivo* after reintroduction into injured myocardium [6, 9]. However, therapeutic potential is limited in the adult heart due to a diminished stem cell pool and modest proliferation and survival ability of CPCs due to aging with a simultaneous up-regulation of senescence and apoptotic markers [11–17]. Therefore, adult CPCs benefit from enhancement prior to adoptive transfer into the damaged myocardium after injury.

Proviral insertion site for the moloney murine leukemia virus (PIM1), a proto-oncogene serine/threonine-protein kinase, belongs to PIM kinase family. PIM1 is highly expressed in bone marrow, tumor cells and fetal heart [18] and is involved in many signaling pathways, mostly related to anti-cell apoptosis and cell cycle regulation [18]. Both human CPCs and mCPCs transduced with *PIM1* significantly enhanced proliferation and survival characteristics *in vitro* and show myocardial functional improvement *in vivo* as after adoptive transfer in rodent models [19, 20].Furthermore, PIM1 modified CPC conferred significant improvement in a preclinical swine infarction model following adoptive transfer delivery [21] highlighting future use of engineered CPCs in clinical trials. PIM1 also acts as a downstream target of cardioprotective pathway in CMs survival [22–25]. PIM1, therefore, serves as an ideal cardioprotective signaling agent and has proven efficacy for stem cell-based therapeutic treatment for heart disease.

Minicircle (MC) vectors are non-viral plasmids with no bacterial backbones or antibiotic resistant genes that serve as an alternative approach to viral-based delivery systems. MC is superior to regular plasmids derived from bacterial DNA that causes transcriptional silencing of the transgene *in vivo* [3, 26, 27]. Additionally, production of antibiotic resistant genes may lead to an altered gene expression profile in the cells [4, 28–30]. Thus, MC is a preferred alternative DNA vector *in vivo* compared to conventional plasmids. MC is also shown to have between 13 to 50 times higher expression of delivered genes compared to standard plasmid delivery [31–33]. For example, Stenler group used gene delivery by MC to mouse cardiac muscle and demonstrated a 1.9 fold higher expression when compared to phVEGF165 plasmid at 7 days post-injection *in vivo* [34]. Huang also demonstrated MC delivered gene expression in the heart for more than 12 weeks and mice with MI and treated with therapeutic genes by MC injections were found to have a higher ejection fraction (EF) (51.3±3.6%) compared to saline group (30.5±2.8%) at 4 weeks post injury and injection [35]. Therefore, experiments were initiated to use MC as a non-viral DNA vector for gene delivery of *PIM1*.

Combining *PIM1*, a pro-survival, cardioprotective gene with MC could generate an enhanced approach for treating MI. We therefore hypothesized that *PIM1* could successfully be delivered through minicircle (*PIM1*-MC) into the myocardium, and enhance cardioprotection post-MI for sustainable recovery after injury. Experiments demonstrate the *in vitro* integration of *PIM1*-MC into mCPCs (*PIM1*-mCPCs) and functional attributes of these cells such as survival and proliferation in response to acute stress. Studies then focus on the *in vivo* integration of *PIM1*-MC into cells within the border zone of an infarcted heart. Results demonstrate *PIM1*-MC has potential to serve as a novel therapeutic treatment for MI.

## Materials and methods

All aspects of this study (*PIM1*-minicircle as a therapeutic treatment for myocardial infarction) involving animal subjects were reviewed and fully approved by the Institutional Animal Care and Use Committee and Office of Laboratory Animal Welfare committees of San Diego State University. Anesthesia was administered for surgical procedures using 1–2.5% isoflurane. Buprenorphine analgesics were used to minimize animal suffering and distress.

### Vector construction

*PIM1* coding sequence (1 kb) was subcloned into the parental plasmid (PP) (10 kb) through EcoRI restriction enzyme digestion and transformed into *E*.*coli* strain ZYCY10P3S2T (System Biosciences), generating *PIM1 turbo gfp* (*PIM1*-PP) for PIM1 overexpression. *Turbo gfp* reporter (*gfp*-PP) was used as MC control. In the *PIM1*-PP, *PIM1* was driven by a cytomegalovirus (CMV) promoter.

### MC production

Protocol for MC production (System Biosciences, Palo Alto, CA) was used in this study with minor modifications. Briefly, one single colony of *E*.*coli* with PP by T-streak was amplified in 2 mL LB solution contains 50 μg/mL kanamycin at 37°C, 250 rpm for 1 hour. Then, specific volume (inoculate volume per 400 mL culture = 0.4/ OD_600_ μL) of cultures were amplified in fresh 400mL terrific broth with 50 μg/mL kanamycin at 30°C, 250 rpm for 16 hours. The OD_600_ was measured by eppendorf biophotometer and kept between 4 and 6 because less grow or over grow will affect future MC yield. 400 mL LB and 400 μL 20% L-arabinose (Sigma) were added to the culture and incubated for another 5 hours at 30°C, 250 rpm. The bacteria pellets were thereafter obtained by centrifugation at 0°C, 5000 rpm for 25 minutes. Plasmid purification was conducted using Nucleo Bond Xtra plasmid purification Kit (Macherey-Nagel, Bethlehem, PA) per manufacture specification.

### Enzyme digestion and electrophoresis

0.5 μg Plasmid DNA was mixed with 1 μL 10 X Cut Smart Buffer (BioLabs, Ipswich, MA), 10 U EcoRI-HF (BioLabs, Ipswich, MA) and molecular grade water filled up to a total 10μL reaction and incubated in 37°C water bath for 10 minutes. Digestion products were subjected in a 1% standard agarose gel with 0.2 μg/μL ethidium bromide by electrophoresis. *PIM1* and MC DNA fragments were confirmed by Typhoon 9410 (GE Healthcare, Little Chalfont, UK).

### Cell culture and transfection

mCPCs were isolated by our lab as previous described [22]. 40,000 mCPCs, passage 12 (p.12), were plated per well in a 6-well plate with DMEM-F12 Mix medium (10% Embryonic Stem Cell Qualified Fetal Bovine Serum (ES FBS), 1% Penicillin/Streptomycine/L-Glutamine, 0.01% Leukemia Inhibitory Factor, 0.2% Insulin Transferrin Selenium, 0.02% Epidermal Growth Factor, 0.02% Basic Fibroblast Growth Factor) and grown at 37°C incubator with 5% CO_2_. Transfection system was optimized for FuGene6 (Promega, Madison, WI) reagent amounts (0–7.5 μL), cell confluency (40%, 60%, 80%), reagent: DNA ratio (6:1, 3:2, 3:1) and single vs. double transfections separately. Transient transfection was done with 1 μg *PIM1*-MC on 2 continuous days at a 3:1 (reagent: DNA) ratio. *Gfp*-MC transfection was done in the same way. 48 hours after transfection, cells were sorted by a fluorescence-activated cell sorting (FACS) Aria Cell Sorter (BD Bioscience, San Diego, CA) to collect GFP^+^ mCPCs. These cells were then plated and rested for 24 hours before proliferation and survival assays.

### Immunoblotting

48 hours post MC transfection, mCPCs samples were collected in 1X sodium dodecyl sulfate (SDS) sample buffer with protease and phosphatase inhibitors. Cell lysates were boiled for 5 minutes and loaded into electrophoresis gels or stored at -80°C.

For *in vivo* heart sample analysis (see surgery conditions below), female FVB mice were sacrificed by cervical dislocation. Hearts were rinsed in 1X cold PBS and mixed with ice-cold isolation buffer (sucrose (70 mM (millimolar)), mannitol (190 mM), HEPES solution (20 mM), and EDTA solution (0.2 mM) in de-ionized water) and beads. The mixture was crushed at 4°C for 5–10 minutes and spun at 12,000 rpm for 1 minute to obtain the supernatants. Heart lysates were diluted in 1X SDS sample buffer.

Proteins were loaded into 4–12% Bis-Tris gel (Thermo Fisher Scientific, Waltham, MA) and run in 1X MES SDS running buffer (Thermo Fisher Scientific, Waltham, MA) at 150 V for 1.3 hours on an electrophoresis apparatus (Invitrogen, Carlsbad, CA). Separate proteins were transferred to a polyvinylidene difluoride membrane in 1X transfer buffer (Thermo Fisher Scientific, Waltham, MA) then blocked with 5% dry milk in 1X TBST (50mmol/L Tris-HCl (pH 7.6)/ 150 mMNaCl/ 0.1% Tween 20) for 1 hour. After blocking, membrane was incubated with primary antibodies consisted of PIM1 anti-mouse (Zymed, 1:1000), turbo GFP anti-rabbit (Invitrogen, 1:1000) and TUBULIN anti-rat (CST, 1:1000) or GAPDH anti-goat (Cheicon, 1:1000) at 4°C overnight. The next day, after washing with 1X TBST four times, the membrane was incubated with secondary antibodies (donkey anti-goat Cy5 (Life technology) or donkey anti-rat Cy5 (Jackson ImmunoResearch), donkey anti-mouse Cy3 (Jackson ImmunoResearch) and donkey anti-rabbit 488 (Jackson ImmunoResearch), 1:5000) at room temperature for 2 hours. After washing three times, the membrane was scanned with a Typhoon 9410 (GE Healthcare, Little Chalfont, UK).

### CyQuant assay

Cell proliferation was done by CyQuant assay based on the cellular DNA content measurement though binding of a fluorescent dye. 24 hours after sorting for GFP^+^ cells, 500 cells/well of *gfp*-MC or *PIM1*-MC transfected mCPCs (*gfp*-mCPCs or *PIM1*-mCPCs) were plated in triplicate into a 96-well tissue culture plate in a total volume of 100 μL/well, and CyQuant dyes (Invitrogen) was used at a 1:1 ratio to determine the DNA content at day 0, day 1, day 3 and day 5 post plating. Nontransfected mCPCs cells were used as a negative control. Fluorescent was measured by a SpectraFluro (Tecan, Männedorf, Switzerland).

### Cell survival assay

FACS was used to test cell survival upon different treatment groups and cell death induced by H_2_O_2_. 60,000 GFP^+^*gfp*-mCPCs or *PIM1*-mCPCs per well were transferred into a 6-well tissue culture plate, and switched to low serum (2% ES FBS) DMEM-F12 media overnight. After the overnight serum starvation, H_2_O_2_ was added to the media to reach a concentration of 40 μM for 4 hours treatment. Cells were then collected for FACS to test cell death using Annexin V (AnV) (BD Pharmingen) and propidium iodide (PI) (Life Technology) staining.

### Myocardial infarction and injection

MI was carried out in ten-week old female FVB mice under 1–2.5% isoflurane anesthesia and induced MI by left anterior descending artery (LAD) ligation. Immediately after infarction injury, injections with PBS (5 μL per injection 3 injections total per mouse), *gfp*-MC, or *PIM1*-MC (5 μL per injection 3 injections total 100 μg of DNA per mouse) plus lipophilic tracers-Di l (0.025 μg/μL) were introduced to the pre-ischemic border along the infarcted region. Sham mice were performed by opening and closing the chest of the mouse without injection of any substance into the heart. Buprenorphine analgesics were used both pre-surgery and 16 hours post-surgery to minimize animal suffering and distress. All the surgery animals were put on the heating pad and monitored every 24 hours until 7 days then moved to the cage shelf. All the animals were under the regular husbandry environment, which includes housing with shredded corncobs bedding layer, regular mouse chow and water and up to 5 female mice per cage.

### Animal subject

All animal studies were approved by the San Diego State University Institutional Animal Care and Use Committee and Office of Laboratory Animal Care.

### Echocardiography

VisualsonicsVevo 2100 system (Visualsonics, Ontario, Canada) was used for echocardiography (echo) to evaluate heart function after MI and injections. Animals were anesthetized under 0.5–1.5% isoflurane and then echoed on a heated pad with the heart rate between 550 and 600 beating per minute. Ejection Fraction (EF) and Fractional Shortening (FS) were acquired using standard B-mode image acquisitions of left ventricular long axis images. Left ventricle anterior wall thickness (LVAW) was measured for wall thinning along a parasternal short-axis view by M-mode. EF at 2 days post infarction/injection was used to check the infarction size. Echo was performed every week up to 4 weeks for cardiac functional measurement. All measurements were analyzed by VisualsonicsVevo 2100 software (Visualsonics, Ontario, Canada).

### Immunofluorescence

Mice were sacrificed under chloral hydrate sedation before heart retroperfusion. Retroperfused hearts were removed from the chest cavity and placed in 10% formalin overnight, followed by 70% ethanol and processing for paraffin embedding using an automated tissue processor ASP 300 (Leica, Wetzlar, Germany). Hearts samples were then processed and sectioned at 6 mm for slides by RM 2245 (Leica, Wetzlar, Germany). Heart sections were deparaffinized, and antigens were retrieved in 1 mM citrate (pH 6.0), followed by 1 hour blocking in 1X TNB (pH 7.5 Tris-HCl (100 mM), NaCl (150 mM), Bovine serum albumin (0.36%) in de-ionized water). Primary antibodies were incubated overnight at 4°C. Slides were washed in 1X TN (Tris/NaC1) three times for 5 minutes followed by secondary antibodies incubation for 2 hours at room temperature. Subsequent tyramide amplification for GFP was performed as necessary. Samples were washed after secondary antibodies in 1X TN, three times for 5 minutes, followed by a final wash containing 4, 6-diamidino-2-phenylindole (DAPI) at 1:10,000 dilution for 10 minutes to stain for nuclei. Primary antibodies used: GFP anti-rabbit (Invitrogen 1:500), myosin light chain 2 (MyL2) anti-goat (Santa Cruz, 1:50), GFP required tyramide amplification. Second antibodies used: donkey anti-goat Cy5 (Life technology) and donkey anti-rabbit 488 (Jackson ImmunoResearch), 1:2000. In this experiment, more than 400 cells were counted per heart section, and 4–6 sections were checked per heart.

### Statistical analysis

Two groups comparison was done by student’s t-test and more than two groups were done by one-way ANOVA with Tukey’s post-test. P value less than 0.05 was considered statistically significant. Error bars represent standard error of the mean (SEM). All the statistical analysis was performed using GraphPad Prism 5 software.

## Results

### Successful insertion of *PIM1* fragment

**Fig 1:**
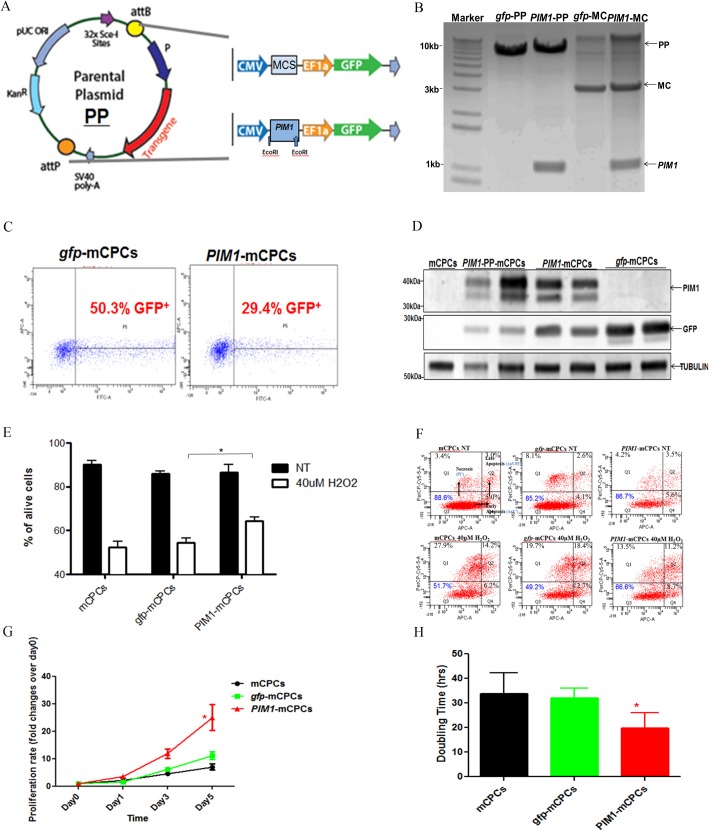
Characterization of *PIM1*-mCPCs. A: *Gfp*-MC and *PIM1*-MC parental plasmids (PP) structures. B: Electrophoresis of PP and MC after EcoRI enzyme digestion. C: Percentage of GFP^+^ mCPCs after MC transfection as measured by FACS. D: PIM1 overexpression in PIM1-mCPC lysate measured by immunoblotting. E: Quantification of cell survival assays. F: Representative cell survival assay by FACS analysis. G: Proliferation rate by CyQuant assay. H: Doubling time by CyQuant assay. NT (no treatment). All error bars are SEM; n = 3 (E and G). *P<0.05, ***P<0.001 vsgfp-mCPCs group by one-way ANOVA.

During MC production, PP was cut at attB and attP sites and formed a smaller plasmid-MC and bacteria backbone (Fig 1A). *PIM1* was subcloned into PP through multiple cloning sites (MCS) for PIM1 overexpression (*PIM1*-PP). MC contains a *turbo gfp* (*gfp*-PP) and was used as a transfection and injection control (Fig 1A). *PIM1* fragment was successfully inserted in both PP and MC particles, shown by electrophoresis after EcoRI digestion (Fig 1B). *PIM1* (1 kb) was detected only in *PIM1*-MC and *PIM1*-PP, but not in *gfp* controls. MC bands (3 kb) were detected in *PIM1*-MC and *gfp*-MC, but not in any PP particles, which confirmed the successful production of MC particles (Fig 1B).

### Transfection conditions optimization

The MC transfection was optimized in a 6-well plate and FuGene6 (0–7.5 μL) reagent was non-toxic on mCPCs when 40,000 cells are initially plated and rested for 4 hours before the experiment begins (data not shown). The optimized transfection condition of mCPCs was serum starvation overnight followed by two 24-hour transfections of 1 μg per 24 hours. The efficiency was 29.4±3.7% with *PIM1*-MC and 50.3±4.2% with *gfp*-MC, as determined by GFP positivity by FACS (Fig 1C). This transfection efficiency correlated with the highest cell survival (data not shown). GFP and PIM1 overexpression in mCPCs were confirmed by immunoblot analysis. PIM1 bands were expressed in *PIM1*-MC and *PIM1*-PP transfected mCPCs, and GFP band was detected in all PP and MC transfected cells (Fig 1D).

### PIM1 enhances mCPCs proliferation and survival by MC delivery

All GFP^+^ cells were sorted by FACS to provide a high purity system for cell proliferation and survival assays. Previous papers also suggested a MC persistence of at least 14 days in transfected cells and 90 days *in vivo* heart injection [35].H_2_O_2_ is a standard method to induce cellular oxidative stress and, based upon dose titration, 40 μM for 4 hours was the optimal dose for inducing cell death that could be mitigated by intervention (S1A Fig). All groups started with 85%-90% viable cells before treatment. *PIM1*-mCPCs remained viable at 66.6±1.8% after the treatment, while *gfp*-mCPCs and non-transfected mCPCs showed a lower survival rate (49.2±2.7% and 51.7±2.1%, respectively) (Fig 1E and 1F). CyQuant assay to measure cell proliferation is widely used in this lab [21–23] and is verified using hemocytometer cell counting technique (S1B Fig). *PIM1*-mCPCs exhibited an increase in proliferation from day one to three (1.9±0.1 fold change, P = 0.0753) and reached a significant difference at day 5 (2.2±0.2 fold change, P<0.05) compared to *gfp-*mCPCs and mCPCs. *Gfp*-mCPCs had no significant difference in proliferation rate up to five days compared to mCPCs (Fig 1G). Significant smaller doubling time of *PIM1*-mCPCs (19.8±6.0 hours) was also shown compared to *Gfp*-mCPCs(32.1±2.4 hours) and mCPCs (33.7±8.8 hours) (Fig 1H, P<0.05). Collectively, these data showed *PIM1*-MC enhance mCPCs proliferation and survival in response to oxidative stress caused by hydrogen peroxide.

### PIM1 is successfully delivered and integrated *in vivo*

**Fig 2:**
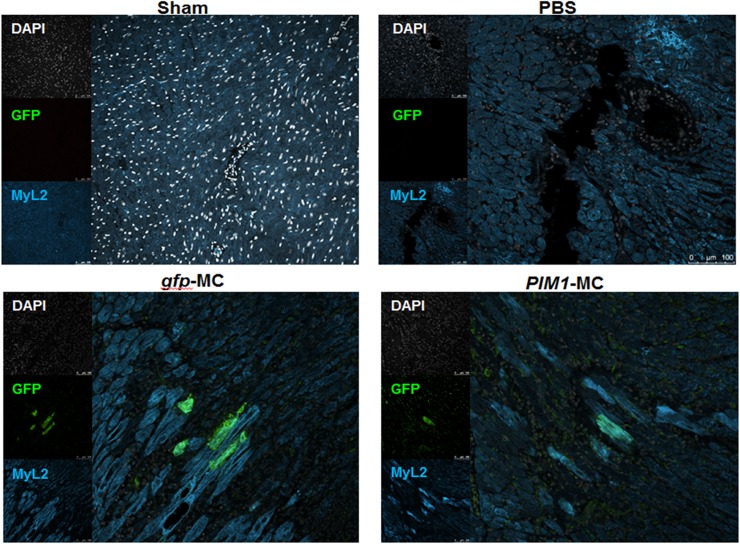
Successful delivery and integration of MC *in vivo*. Representative confocal images of border zone GFP^+^ cells in heart section after MC injection at 3 dpi by immunohistochemistry. Green: GFP; white: DAPI; blue: MyL2. Scale bar is 100 μm.

Since *PIM1* modified mCPCs by MC showed better survival and proliferation *in vitro* under stress, *in vivo* characterization of cardiac function post MI and treatment with either placebo, *gfp*-MC or *PIM1-*MC was a logical next step. 100 μg of *PIM1*-MC with 0.375 μg Di l was injected into injury site immediately upon acute MI injury in ten weeks old FVB female mice. No MC or Di l was injected in sham group. Di l was detectable and used for localizing MC injection sites at 3 days post injury (dpi) (S2 Fig). Successful MC delivery was confirmed by GFP^+^ cells detected 3 dpi *in vivo* via immunohistochemistry staining (Fig 2). GFP^+^ cells were found in 2 out of 4 *gfp*-MC injected mice and 1 out of 1 *PIM1*-MC injected mouse (Fig 2). All GFP^+^ cells found were limited to the border zone CMs. 12 GFP^+^ CM were found in *gfp*-MC injected heart and 1 GFP^+^ CM was found in a *PIM1*-MC injected heart (Fig 2). The GFP expression demonstrates the MC DNA injected *in vivo* was able to integrate into surrounding cells and could be transcribed and translated. Whole heart lysates were processed for immunoblotting to quantitate PIM1 and GFP expression. PIM1 and GFP proteins were not detected through western blot (S3 Fig).

### *PIM1*-MC demonstrates moderate improvement short-term after myocardial infarction

**Fig 3:**
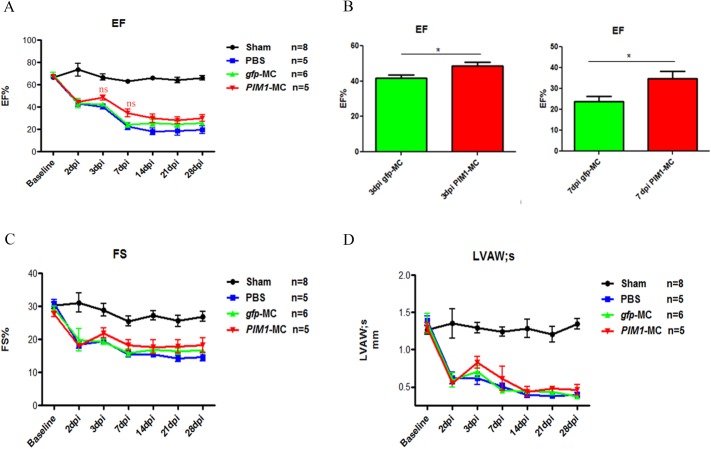
*PIM1*-MC has a moderate cardioprotection short-term after myocardial infarction. A: Longitudinal assessment of EF (%) up to 28 days. B: EF (%) of *gfp*-MC and *PIM1*-MC groups at 3dpi and 7dapi. C: Longitudinal assessment of FS (%) up to 28 days. D: Longitudinal assessment of LVAWs up to 28 days. Sample sizes of 5–8 mice per group. ns (not significant), *P<0.05, ***P<0.001 vs. *gfp*-MC control group. A, C and D by one-way ANOVA. B by student t-test.

Potential therapeutic efficacy of *PIM1*-MC naked DNA injection on heart injury was conducted in ten-week old female FVB mice. Left anterior descending artery (LAD) was ligated to induce MI and 100 μg *PIM1*-MC or *gfp*-MC was immediately injected at the border zone. Infarction consistency by LAD ligation among each group was confirmed by parasternal echocardiography as a similar drop in EF at 2dpi. Mice with mild (EF% >55%) or severe drop (EF% < 35%) at 2 dpi were excluded; remaining mice, with a similar function loss, were included in this study (n = 5–8 mice per group). During the initial post-surgery period, *PIM1*-MC injected mice displayed a higher EF value at 3 dpi and 7 dpi compared to *gfp*-MC and PBS injected groups (P = 0.0625 and P = 0.0562 using one-way ANOVA, respectively) (Fig 3A). When comparing *gfp*-MC and *PIM1*-MC group at 3 dpi and 7 dpi by t-test, *PIM1*-MC showed significant (P < 0.05) higher EF (Fig 3B). As EF continued to decrease in all MI mice, a plateau was reached at 14 dpi. *PIM1*-MC injected mice remained at a slightly higher EF at all time points compared to sham and *gfp*-MC injected mice. By 28 dpi, *PIM1*-MC injected mice remained at a better EF and FS level in comparison to all other MI groups (Fig 3A). A similar tendency between these three groups was also observed in FS and left ventricular anterior wall thickness at systole (LVAWs) (Fig 3C and 3D). Representative 3 dpi echocardiography images show all MI hearts have dilated ventricles, thinner left ventricular anterior wall thickness (LVAW), and loss of contractility in LVAW, whereas Sham hearts maintained regular ventricular size and normal contractility (S4 Fig). Overall, the *PIM1*-MC protection peaks at 3 dpi and continues to maintain a slower rate of functional decrease over 28 days.

## Discussion

MC is used as a non-viral therapeutic gene delivery to heart muscles for treating ischemic disease recently [34, 35]. Our lab has shown PIM1 enhanced CPCs have a faster proliferation rate and survival rate *in vitro* and improved heart function *in vivo*[22, 23]. In an effort to find a low risk delivery method for therapeutic gene *PIM1*, we tested the potential efficacy of *PIM1* delivery by MC.

In our *in vitro* study, optimal *PIM1*-MC transfection efficiency of 29.4±3.7% was achieved through serum starvation of mCPCs overnight followed by two transfections, spaced 24 hours apart. Using FACS to enrich for MC transfected mCPCs, we recapitulated the previous finding that CPCs enhanced by lentivirus-based *PIM1* delivery proliferate faster compared to *gfp* control group [22]. Similarly to these previous lentivirus-based PIM1 delivery enhanced mCPCs studies, we found *PIM1*-mCPCs enhanced through MC transfection demonstrate higher survival rates under oxidative stress compared to *gfp* controls [22, 24].

*In vivo*, uptake and expression of directly injected DNA is possible in many tissues and organs including heart, skin, liver and muscles [36–40]. Generally, the methods of detection are limited to luciferase or β-gal based assays [36–40]. This study demonstrates the potential for direct immunohistochemical detection of foreign gene expression and localization in the cells. *PIM1*-MC was successfully up taken and expressed in border zone CMs at 3 dpi *in vivo*. This highlights the possibility of a non-cell based therapeutic treatment for MI.

A challenge of this study was in the limited number of GFP^+^ CMs detected in the *gfp*-MC and *PIM1*-MC injected hearts. One factor influencing the detection of GFP is the variable expression of GFP, which diminishes over time [41]. Numerous publications also point out the concern about variable and tissue-specific expression pattern of CMV promoter inducing gene silencing [41–43], which may have also affected the GFP expression. Additionally, the limited GFP^+^ cells in the *PIM1*-MC injected group may have resulted from the different plasmid sizes of *PIM1*-MC (4 kb) and *gfp*-MC (3 kb), as DNA size affects gene uptake *in vivo* [38, 44]. Minicircle transfection efficiency is challenging *in vitro* and provides some insight into the challenges of *in vivo* transfection. One means to offset this challenge and potentially transfect more cells is through a higher concentration of *PIM1*-MC injection.

Simple direct gene injection has many advantages. Non-cell based therapy removes the work for prior cell isolation, culturing and transfection. In addition, DNA direct injection reduces the possible immune response caused by select cell-based therapies. *PIM1*-MC showed moderate cardioprotection short-term after myocardial infarction by EF compared to *gfp*-MC or the PBS group. Although a low efficiency of *PIM1*-MC to fully transfect CMs was found in these studies, cardioprotection may have resulted through paracrine signaling of the CMs with the increased levels of PIM1 to induce an anti-apoptotic response. Long-term tracking of *PIM1*-MC will assist in assessing its therapeutic potential. Increasing the amount of MC DNA injection and/or continuing injections every 1–2 weeks are additional alternative experimental designs to potentially improve *in vivo* efficiency. *PIM1*-MC direct injection nevertheless reveals promising results and warrants future investigation. In conclusion, this study demonstrates the *in vitro* and *in vivo* feasibility and therapeutic potential of *PIM1* delivered by MC.

## Supporting information

S1 Fig Optimization of cell survival and proliferation systems. A:H_2_O_2_ treatment conditions titration by FACS analysis.B: Doubling time comparasion by CyQuant and hemocytometer cell counting. NT (no treatment).
(TIF)

S2 Fig Representative confocal images of 3 dpi Di l tracking for MC injection localization. No Di l or MC injected in Sham group. Red: Dil. Scale bar is 250 μm.
(TIF)

S3 Fig GFP and PIM1 are undetectable in 3 dpi whole heart lysates by immunoblotting. (TIF)

S4 Fig Examples of 3 dpi sham and MI mice images in longitudinal B-mode and M-mode by echo. (TIF)

## References

[1] Ferreira-González I. The Epidemiology of Coronary Heart Disease. Revista Española de Cardiología (English Edition). 2014;67: 139–144. 10.1016/j.rec.2013.10.002 24795124

[2] National Heart, Lung and Blood Institute (NHLBI). Disease statistics. In: NHLBI Fact Book, Fiscal Year 2012.

[3] Roger VL, Go AS, Lloyd-Jones DM, Benjamin EJ, Berry JD, Borden WB, et al Heart Disease and Stroke Statistics—2012 Update: A Report from the American Heart Association. Circulation. 2011;125. 22179539 10.1161/CIR.0b013e31823ac046 PMC4440543

[4] Abraham WT, Smith SA. Devices in the management of advanced, chronic heart failure. Nature Reviews Cardiology. 2012;10(2):98–110. 10.1038/nrcardio.2012.178 23229137 PMC3753073

[5] Daviglus ML, Lloyd-Jones DM, Pirzada A. Preventing Cardiovascular Disease in the 21st Century. American Journal of Cardiovascular Drugs. 2006;6(2):87–101. 16555862 10.2165/00129784-200606020-00003

[6] Bolli R, Chugh A, D'Amario D, Loughran J, Stoddard M, Ikram S, et al Cardiac stem cells in patients with ischaemic cardiomyopathy (SCIPIO): initial results of a randomised phase 1 trial. The Lancet. 2011;378(9806):1847–1857. 10.1016/S0140-6736(11)61590-0 PMC3614010 22088800

[7] Cardiac stem cell therapies for congenital heart diseases. Stem Cell & Translational Investigation Stem Cell TranslInvestig. 2015.

[8] Srivastava D, Ivey KN. Potential of stem-cell-based therapies for heart disease. Nature. 2006;444: 512–512. 10.1038/nature04961 16810246

[9] C. F, M. S, G. G, C. L, S. C, L. P, et al Resident cardiac stem cells. CPDCurrent Pharmaceutical Design. 2011;17(30):3252–3257. 10.2174/138161211797904181 22114897

[10] Beltrami AP, Barlucchi L, Torella D, Baker M, Limana F, Chimenti S, et al Adult cardiac stem cells are multipotent and support myocardial regeneration. Cell. 2003;114: 763–776. 14505575 10.1016/s0092-8674(03)00687-1

[11] Ludke A, Li R, Weisel R-K, Weisel RD. The rejuvenation of aged stem cells for cardiac repair. Canadian Journal of Cardiology. 2014;30(11):1299–1306. 10.1016/j.cjca.2014.03.021 25092405

[12] Sahin E, DePinho RA. Linking functional decline of telomeres, mitochondria and stem cells during ageing. Nature. 2010;464(7288):520–528. 10.1038/nature08982 20336134 PMC3733214

[13] Li S-H, Sun Z, Brunt KR, Shi X, Chen M-S, Weisel RD, et al Reconstitution of aged bone marrow with young cells repopulates cardiac-resident bone marrow-derived progenitor cells and prevents cardiac dysfunction after a myocardial infarction. European Heart Journal. 2012;34(15):1157–1167. 10.1093/eurheartj/ehs072 22507976

[14] Cesselli D, Beltrami AP, D'Aurizio F, Marcon P, Bergamin N, Toffoletto B, et al Effects of age and heart failure on human cardiac stem cell function. The American Journal of Pathology. 2011;179(1):349–366. 10.1016/j.ajpath.2011.03.036 21703415 PMC3175070

[15] Dimmeler S, Leri A. Aging and disease as modifiers of efficacy of cell therapy. Circulation Research. 2008;102(11):1319–1330. 10.1161/CIRCRESAHA.108.175943 18535269 PMC2728476

[16] Zhuo Y, Li S-H, Chen M-S, Wu J, Kinkaid HYM, Fazel S, et al Aging impairs the angiogenic response to ischemic injury and the activity of implanted cells: combined consequences for cell therapy in older recipients. The Journal of Thoracic and Cardiovascular Surgery. 2010;139(5):1286–1294.e2. 10.1016/j.jtcvs.2009.08.052 19931095

[17] Zhang H. Increasing donor age adversely impacts beneficial effects of bone marrow but not smooth muscle myocardial cell therapy. AJP: Heart and Circulatory Physiology. 2005;289(5):H2089–H2096. 16219813 10.1152/ajpheart.00019.2005

[18] Eichmann A, Yuan L, Bréant C, Alitalo K, Koskinen PJ. Developmental expression of Pim kinases suggests functions also outside of the hematopoietic system. Oncogene. 2000;19(9):1215–1224. 10.1038/sj.onc.1203355 10713710

[19] Mohsin S, Khan M, Toko H, Bailey B, Cottage C, Wallach K, et al Human cardiac progenitor cells engineered with Pim-I kinase enhance myocardial repair. Journal of the American College of Cardiology. 2012;60(14):1278–1287. 10.1016/j.jacc.2012.04.047 22841153 PMC3461098

[20] Bachmann M, Möröy T. The serine/threonine kinase Pim-1. The International Journal of Biochemistry & Cell Biology. 2005;37(4):726–730. 15694833 10.1016/j.biocel.2004.11.005

[21] Kulandavelu S, Karantalis V, Fritsch J, Hatzistergos KE, Loescher VY, McCall F, et al Pim1 kinase overexpression enhances ckit+ cardiac stem cell cardiac repair following myocardial infarction in swine. Journalof the American College of Cardiology. 2016. In Press. 10.1016/j.jacc.2016.09.925 PMC5223744 27908351

[22] Fischer KM, Cottage CT, Wu W, Din S, Gude NA, Avitabile D, et al Enhancement of myocardial regeneration through genetic engineering of cardiac progenitor cells expressing Pim-1 kinase. Circulation. 2009;120(21):2077–2087. 10.1161/CIRCULATIONAHA.109.884403 19901187 PMC2787902

[23] Muraski JA, Rota M, Misao Y, Fransioli J, Cottage C, Gude N, et al Pim-1 regulates cardiomyocyte survival downstream of Akt. Nature Medicine. 2007;13(12):1467–1475. 10.1038/nm1671 18037896

[24] Wang Z, Bhattacharya N, Weaver M, Petersen K, Meyer M, Gapter L, et al Pim-1: a serine/threonine kinase with a role in cell survival, proliferation, differentiation and tumor genesis. Journal of Veterinary Science. 2001; 2:167–179. 12441685

[25] Katakami N, Kaneto H, Hao H, Umayahara Y, Fujitani Y, Sakamoto K, et al Role of Pim-1 in smooth muscle cell proliferation. Journal of Biological Chemistry. 2004;279(52):54742–54749. 10.1074/jbc.M409140200 15471855

[26] Levine BL, Humeau LM, Boyer J, MacGregor R, Rebello T, Lu X, et al Gene transfer in humans using a conditionally replicating lentiviral vector. Proceedings of the National Academy of Sciences. 2006;103(46):17372–17377. 10.1073/pnas.0608138103 PMC1635018 17090675

[27] Hu K. Vectorology and factor delivery in induced pluripotent stem cell reprogramming. Stem Cells and Development. 2014;23(12):1301–1315. 10.1089/scd.2013.0621 24625220 PMC4046209

[28] Cunningham-Rundles CPonda P. Molecular defects in T- and B-cell primary immunodeficiency diseases. Nature Reviews Immunology. 2005;5(11):880–892. 10.1038/nri1713 16261175

[29] Hacein-Bey-Abina S. LMO2-associated clonal T cell proliferation in two patients after gene therapy for SCID-X1. Science. 2003;302(5644):415–419. 10.1126/science.1088547 14564000

[30] McCormack MRabbitts T. Activation of the T-cell oncogene LMO2 after gene therapy for X-linked severe combined immunodeficiency. New England Journal of Medicine. 2004;350(9):913–922. 10.1056/NEJMra032207 14985489

[31] Chen Z. Minicircle DNA vectors devoid of bacterial DNA result in persistent and high-level transgene expression in vivo. Molecular Therapy. 2003;8(3):495–500. 12946323 10.1016/s1525-0016(03)00168-0

[32] Darquet A, Rangara R, Kreiss P, Schwartz B, Naimi S, Delaère P, et al Minicircle: an improved DNA molecule for in vitro and in vivo gene transfer. Gene Therapy. 1999;6(2):209–218. 10.1038/sj.gt.3300816 10435105

[33] Narsinh K, Jia F, Robbins R, Kay M, Longaker M, Wu J. Generation of adult human induced pluripotent stem cells using nonviral minicircle DNA vectors. Nature Protocol. 2010;6(1):78–88. 10.1038/nprot.2010.173 PMC3657506 21212777

[34] Stenler S, Andersson A, Simonson O, Lundin K, Chen Z, Kay M, et al Gene transfer to mouse heart and skeletal muscles using a minicircle expressing human vascular endothelial growth factor. Journal of Cardiovascular Pharmacology. 2009;53(1):18–23. 10.1097/FJC.0b013e318194234e 19129741

[35] Huang M, Chen Z, Hu S, Jia F, Li Z, Hoyt G, et al Novel minicircle vector for gene therapy in murine myocardial infarction. Circulation. 2009;120(11_suppl_1):S230–S237. 19752373 10.1161/CIRCULATIONAHA.108.841155 PMC3163107

[36] Manthorpe M, Cornefert-Jensen F, Hartikka J, Felgner J, Rundell A, Margalith M, et al Gene therapy by intramuscular injection of plasmid DNA: studies on firefly luciferase gene expression in mice. Human Gene Therapy. 1993;4(4):419–431. 10.1089/hum.1993.4.4-419 8399489

[37] Hengge U, Walker P, Vogel J. Expression of naked DNA in human, pig, and mouse skin. Journal of Clinical Investigation. 1996;97(12):2911–2916. 10.1172/JCI118750 8675706 PMC507388

[38] Li K, Welikson R, Vikstrom K, Leinwand L. Direct gene transfer into the mouse heart. Journal of Molecular and Cellular Cardiology. 1997;29(5):1499–1504. 10.1006/jmcc.1997.0389 9201634

[39] Sikes M, O'Malley B, Finegold M, Ledley F. In vivo gene transfer into rabbit thyroid follicular cells by direct DNA injection. Human Gene Therapy. 1994;5(7):837–844. 10.1089/hum.1994.5.7-837 7981308

[40] Wolff J, Ludtke J, Acsadi G, Williams P, Jani A. Long-term persistence of plasmid DNA and foreign gone expression in mouse muscle. Human Molecular Genetics. 1992;1(6):363–369. 1301910 10.1093/hmg/1.6.363

[41] Mctaggart R, Feng S. An uncomfortable silence … while we all search for a better reporter gene in adult stem cell biology. Hepatology. 2004;39(4):1143–1146. 10.1002/hep.20192 15057919

[42] Swenson E, Price J, Brazelton T, Krause D. Limitations of green fluorescent protein as a cell lineage marker. Stem Cells. 2007;25(10):2593–2600. 10.1634/stemcells.2007-0241 17615263

[43] Walter I, Fleischmann M, Klein D, Muller M, Salmons B, Gunzburg WH, et al Rapid and sensitive detection of enhanced green fluorescent protein expression in paraffin sections by confocal laser scanning microscopy. The Histochemical Journal. 2000;32:99–103. 10816074 10.1023/a:1004014211408

[44] Jechlinger W. Optimization and delivery of plasmid DNA for vaccination. Expert Review of Vaccines. 2006;5(6):803–825. 10.1586/14760584.5.6.803 17184219

