# Supplementary figures and images for: PIM1-minicircle as a therapeutic treatment for myocardial infarction
Source: PLoS One. 2017 Mar 21;12(3):e0173963. doi: 10.1371/journal.pone.0173963 (PMC5360264; doi:10.1371/journal.pone.0173963)

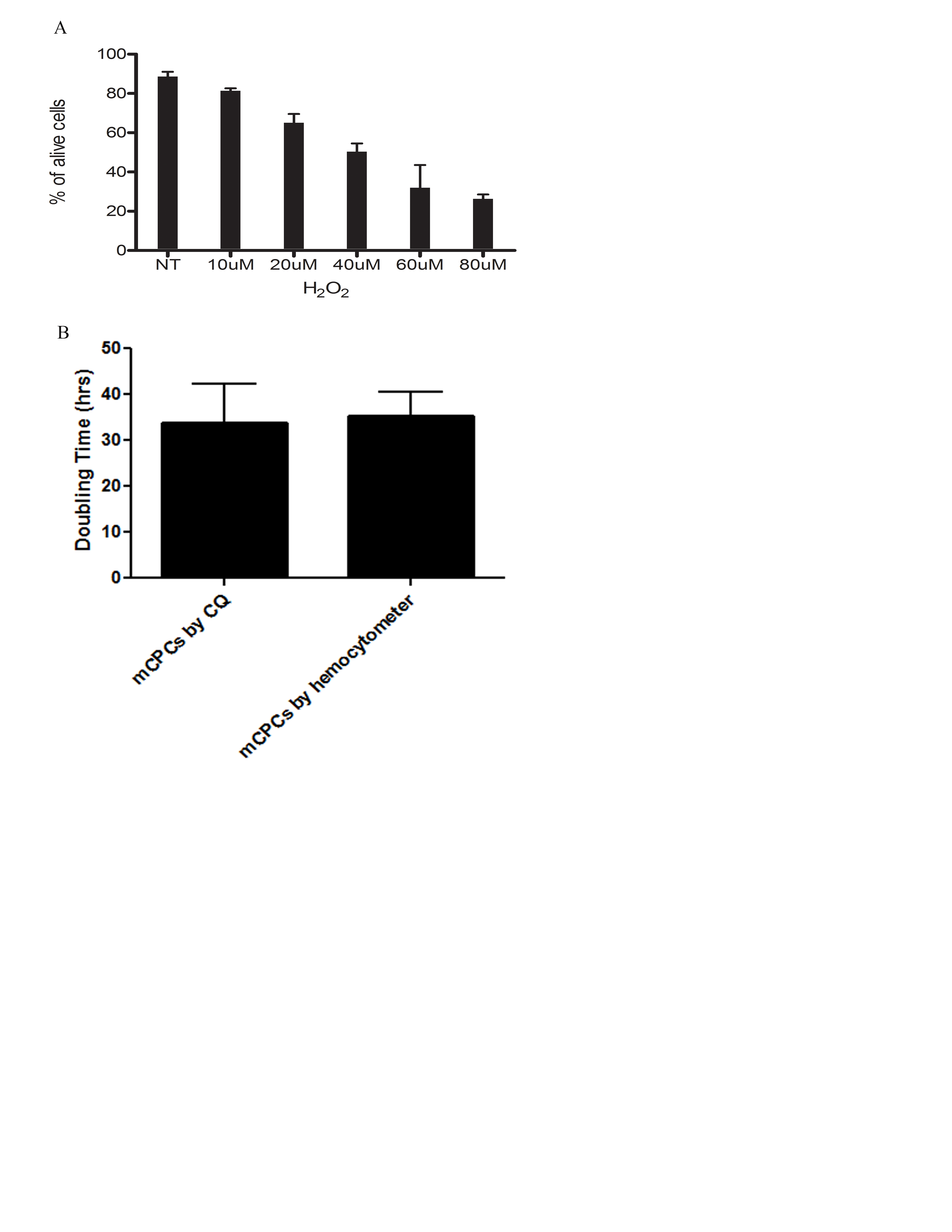

Supplement: S1 Fig — A:H2O2 treatment conditions titration by FACS analysis.B: Doubling time comparasion by CyQuant and hemocytometer cell counting. NT (no treatment). (TIF) [file pone.0173963.s001.tif]

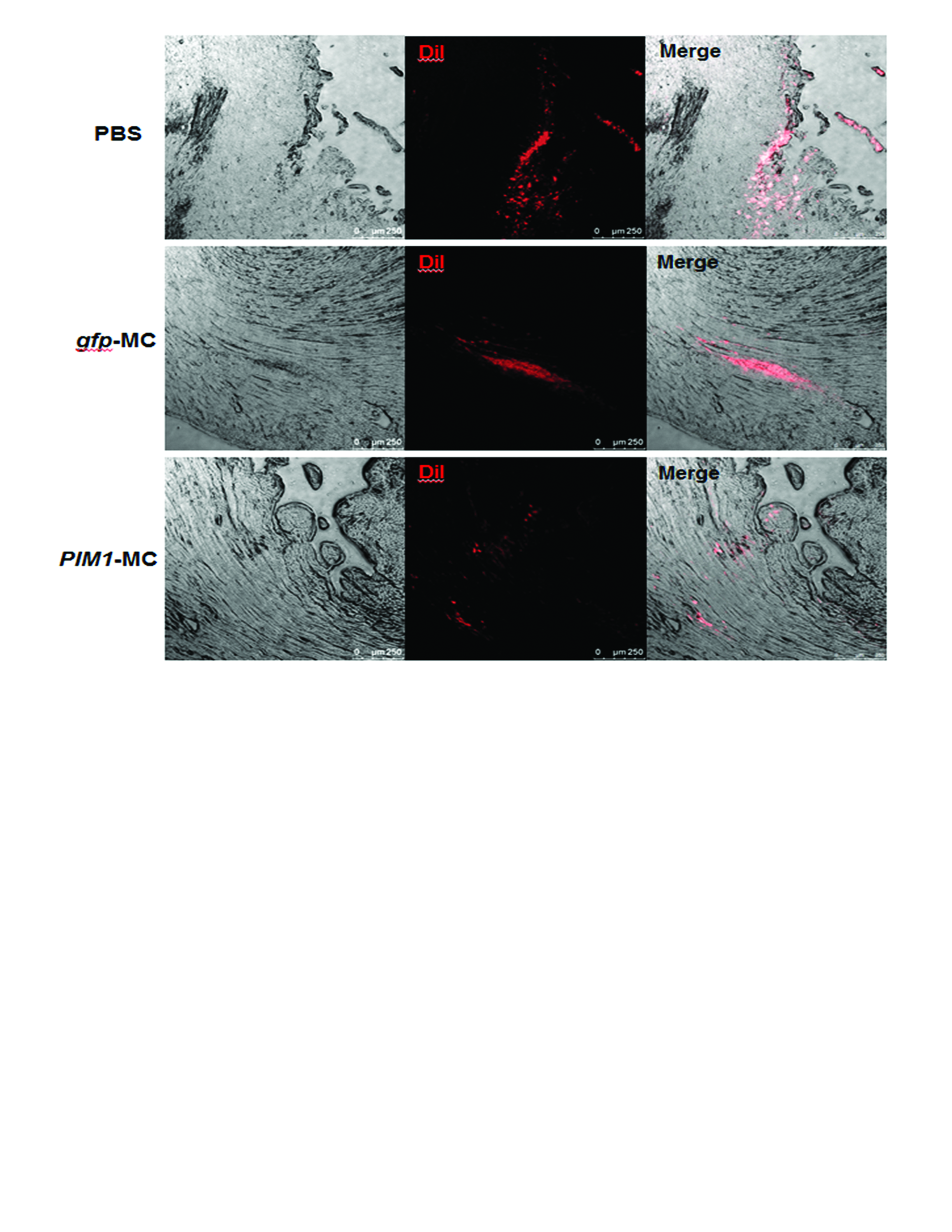

Supplement: S2 Fig — No Di l or MC injected in Sham group. Red: Dil. Scale bar is 250 μm. (TIF) [file pone.0173963.s002.tif]

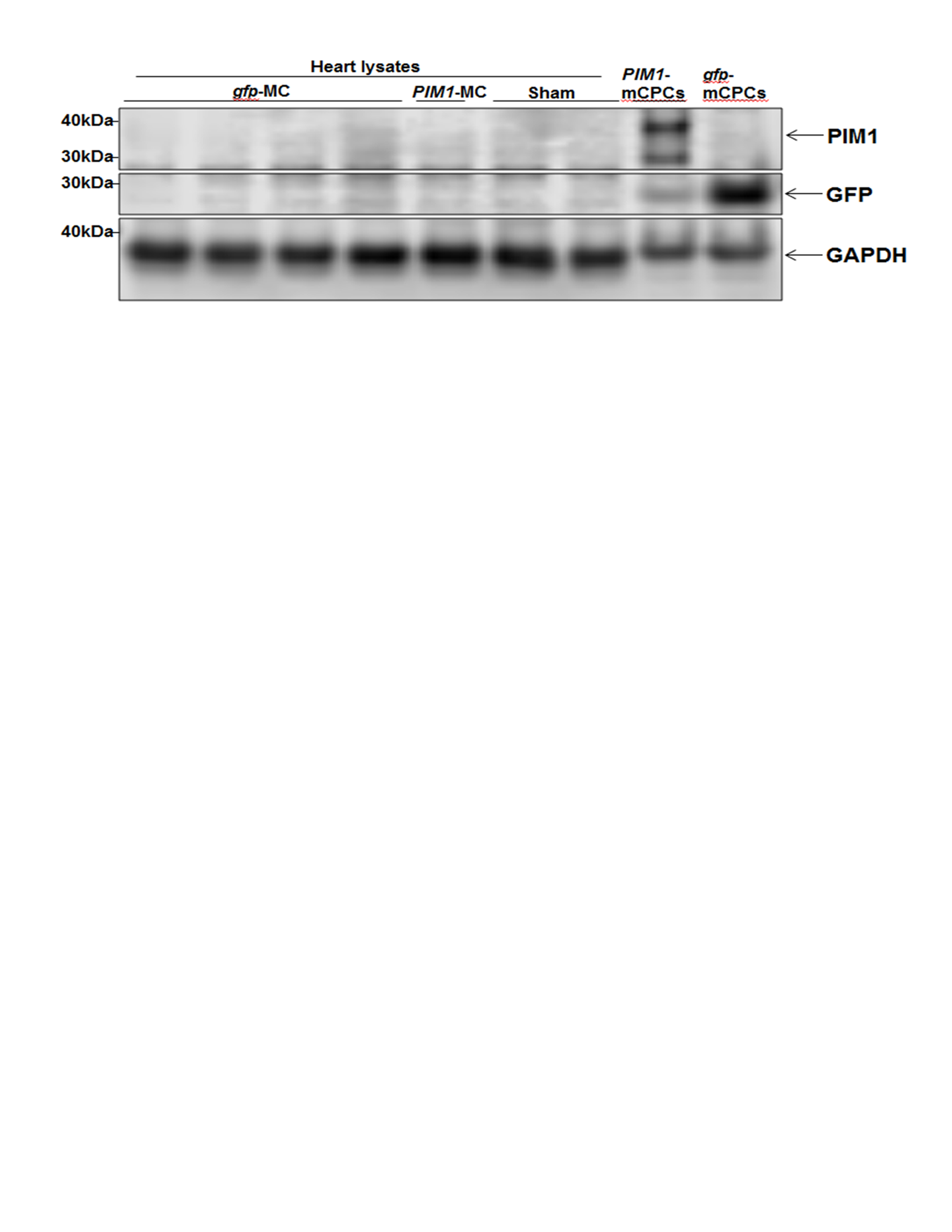

Supplement: S3 Fig — (TIF) [file pone.0173963.s003.tif]

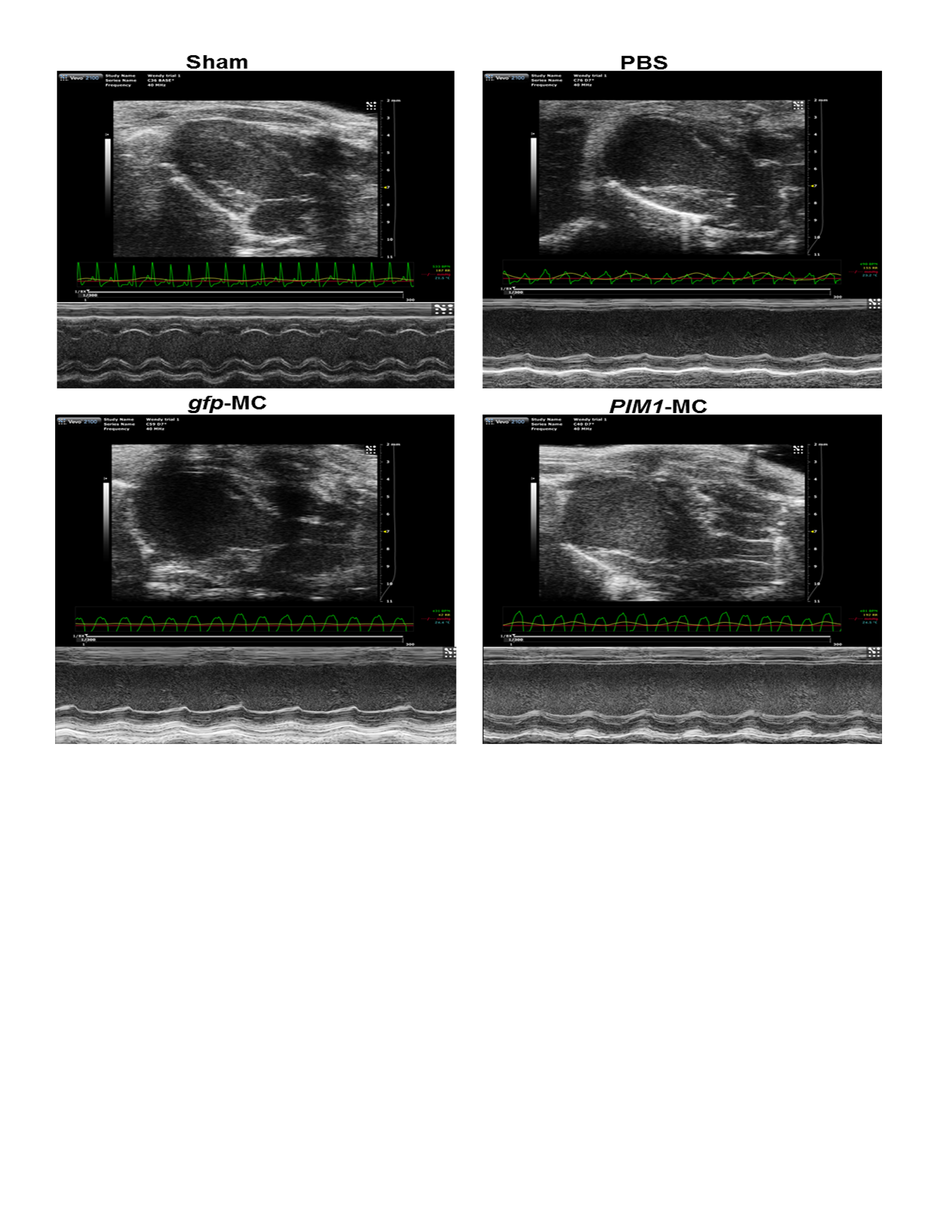

Supplement: S4 Fig — (TIF) [file pone.0173963.s004.tif]
